# Association between tranexamic acid administration and mortality based on the trauma phenotype: a retrospective analysis of a nationwide trauma registry in Japan

**DOI:** 10.1186/s13054-024-04871-w

**Published:** 2024-03-19

**Authors:** Jotaro Tachino, Shigeto Seno, Hisatake Matsumoto, Tetsuhisa Kitamura, Atsushi Hirayama, Shunichiro Nakao, Yusuke Katayama, Hiroshi Ogura, Jun Oda

**Affiliations:** 1 Department of Traumatology and Acute Critical Medicine, Osaka University Graduate School of Medicine, 2-15 Yamada-oka, Suita City, Osaka Japan; 2 Department of Bioinformatic Engineering, Graduate School of Information Science and Technology, Osaka University, 1-5 Yamada-oka, Suita City, Osaka Japan; 3 Division of Environmental Medicine and Population Sciences, Department of Social and Environmental Medicine, Osaka University Graduate School of Medicine, 2-2 Yamada-oka, Suita City, Osaka Japan; 4 Public Health, Department of Social Medicine, Osaka University Graduate School of Medicine, 2-2 Yamada-oka, Suita City, Osaka Japan

**Keywords:** Tranexamic acid, Clinical phenotype, Nationwide cohort, Blunt trauma

## Abstract

**Background:**

In trauma systems, criteria for individualised and optimised administration of tranexamic acid (TXA), an antifibrinolytic, are yet to be established. This study used nationwide cohort data from Japan to evaluate the association between TXA and in-hospital mortality among all patients with blunt trauma based on clinical phenotypes (trauma phenotypes).

**Methods:**

A retrospective analysis was conducted using data from the Japan Trauma Data Bank (JTDB) spanning 2019 to 2021.

**Results:**

Of 80,463 patients with trauma registered in the JTDB, 53,703 met the inclusion criteria, and 8046 (15.0%) received TXA treatment. The patients were categorised into eight trauma phenotypes. After adjusting with inverse probability treatment weighting, in-hospital mortality of the following trauma phenotypes significantly reduced with TXA administration: trauma phenotype 1 (odds ratio [OR] 0.68 [95% confidence interval [CI] 0.57–0.81]), trauma phenotype 2 (OR 0.73 [0.66–0.81]), trauma phenotype 6 (OR 0.52 [0.39–0.70]), and trauma phenotype 8 (OR 0.67 [0.60–0.75]). Conversely, trauma phenotypes 3 (OR 2.62 [1.98–3.47]) and 4 (OR 1.39 [1.11–1.74]) exhibited a significant increase in in-hospital mortality.

**Conclusions:**

This is the first study to evaluate the association between TXA administration and survival outcomes based on clinical phenotypes. We found an association between trauma phenotypes and in-hospital mortality, indicating that treatment with TXA could potentially influence this relationship. Further studies are needed to assess the usefulness of these phenotypes.

**Graphical abstract:**

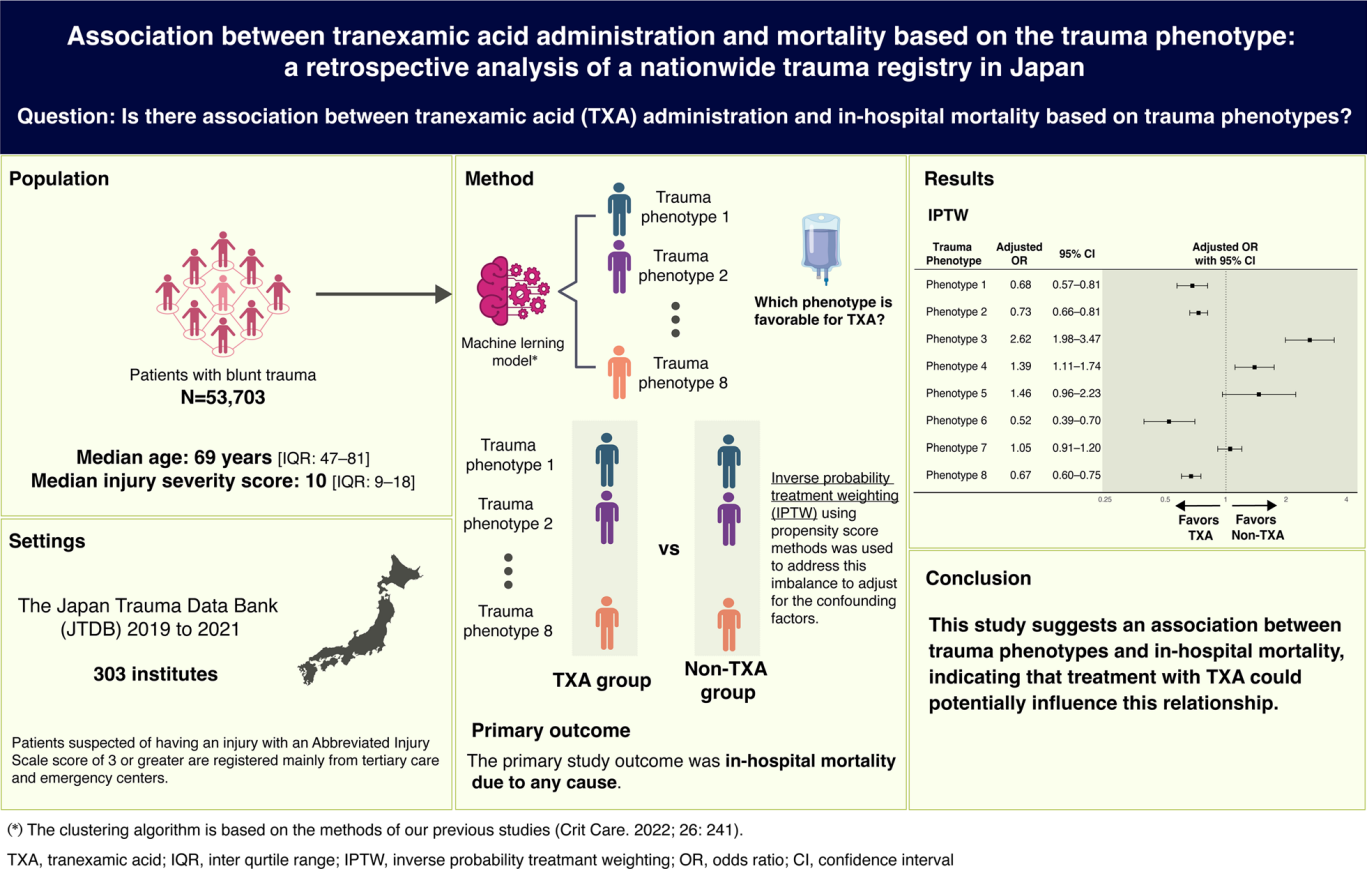

**Supplementary Information:**

The online version contains supplementary material available at 10.1186/s13054-024-04871-w.

## Background

Despite global efforts to standardise trauma care and improve treatment outcomes, approximately 4.5 million trauma-related deaths occur annually worldwide [1]. Most preventable early trauma deaths are due to haemorrhage [2]. Early trauma-induced coagulopathy (TIC) exacerbates this by contributing to acute bleeding, associated shock, ischaemia–reperfusion injuries, thrombotic complications, and, in severe cases, hyperfibrinolysis, further promoting haemorrhage [3]. Tranexamic acid (TXA), a synthetic derivative of the amino acid—lysine—is an antifibrinolytic that inhibits lysine-binding sites on plasminogen [4]. In the international randomised controlled trials (RCTs)—CRASH-2 [5, 6] and CRASH-3 [7]—TXA demonstrated the potential to improve outcomes in patients with traumatic bleeding and mild-to-moderate traumatic brain injuries. Therefore, early management of bleeding and coagulation abnormalities in trauma care is recommended [8]. The recent PATCH-Trauma trial conducted on adult patients with severe trauma revealed that early prehospital TXA administration in suspected cases of TIC was not beneficial for survival with functional outcomes at 6 months post-injury. However, secondary analyses suggested potential benefits for survival at 24 h and 1-month post-injury [9]. A meta-analysis including RCTs and observational studies that evaluated TXA efficacy in patients with traumatic injuries and brain injuries aged ≥ 15 years suggested that TXA significantly reduced 1-month mortality rates compared to controls, indicating potential benefits across various patient groups [10]. However, some studies lacked data on age, sex, trauma severity, and comorbidities, resulting in heterogeneity among the study groups; thus, patients most suited for TXA administration remained unclear to clinicians. Given the high heterogeneity among patients with trauma, heterogeneity of the treatment effect might exist at a more granular level, even among severely injured patients targeted in previous RCTs [11]. Additionally, less severely injured patients may benefit from TXA administration.

The precision medicine approach, which involves identifying latent subgroups (phenotypes) of diseases with high heterogeneity, such as sepsis and acute respiratory distress syndrome (ARDS), has recently been shown to enhance the identification of more homogenous groups. This approach deepens the understanding of the pathophysiological mechanisms and contributes to the identification of more effective targets for specific treatments through appropriate stratification [12–14]. This strategy, which identifies subgroups that are more likely to respond to treatment, is called predictive enrichment; it aims to identify specific sub-phenotypes within diseases, reducing heterogeneity and pinpointing subgroups that may respond more favourably to targeted therapeutic interventions [15]. For instance, although an RCT of simvastatin in ARDS did not demonstrate a significant difference in the primary outcome [16], a secondary analysis revealed a beneficial effect in certain phenotypes [17]. This finding underscores the potential of such approaches in enhancing treatment efficacy through refined patient stratification.

Our previous study identified different clinical phenotypes based on the information gathered early in trauma care [18]. The study identified 8 distinct trauma phenotypes (sub-classified into 11 phenotypes) with 14 variables using statistical machine learning techniques. We hypothesised that these subgroup differences would result in heterogenous treatment effects of TXA among trauma phenotypes. Determining whether TXA administration is preferred, based on the trauma phenotype in all patients with blunt trauma, could aid in clinical decision-making and potentially fill the knowledge gap regarding optimal TXA administration. Therefore, this study was conducted using nationwide cohort data to evaluate the association between TXA administration and the survival outcome based on the trauma phenotype in patients with blunt trauma with survival as the outcome measure.

## Methods

### Study aim, design, and settings

We conducted a retrospective analysis using data from the Japan Trauma Data Bank (JTDB) to clarify the association between TXA administration and the survival outcome based on the trauma phenotype. The JTDB registers patients who are assumed to have abbreviated injury scale (AIS) 3 or higher trauma and are transported to hospital (see Additional file 1 for information about the database). We reviewed relevant data from the JTDB between 2019 and 2021 for this study. The therapeutic intervention in this study was the administration of TXA.

### Selection of participants

Patient selection was based on previously identified trauma phenotypes [18], and all patients with blunt trauma registered with the JTDB were included. We excluded patients with non-direct transportation, those who experienced cardiac arrest upon hospital arrival (with heart rate = 0 or systolic blood pressure = 0), and those with injury severity scores (ISSs) of 75. Additionally, patients with missing data on age or sex or unknown discharge outcomes were excluded.

### Data collection

The patients were followed up until discharge or death. The data collected included age, sex, medical history, mechanism of injury, vital signs upon arrival, AIS codes [19], transfusion history, lactate levels at admission, and outcomes.

Trauma severity was assessed using the ISS [20], revised trauma score [21], and survival probability based on the trauma and ISS method [22]. Definitions of one unit of transfused packed red blood cells vary among Japan, the United States of America, and the United Kingdom and are approximately 140 mL, 250 mL, and 280 mL, respectively. In this study, the volume of transfusion was reported in Japanese units. Information on treatment interventions, such as medication, surgery, and interventional radiology, as well as information on functional outcomes (Glasgow Outcome Scale [23]), was also collected.

### Definitions and outcome

The primary study outcome was in-hospital mortality due to any cause. This study was conducted in accordance with the Declaration of Helsinki and approved by the Ethics Committee of Osaka University (IRB approval number 16260–4).

### Statistical analysis

Patient characteristic data are expressed as mean values (with standard deviations) or medians (with interquartile ranges), as appropriate. The balance of covariates at baseline was assessed using the absolute standardised mean difference (ASMD), with values > 0.1 usually indicating an imbalance [24]. However, to ensure no critical information was lost, we chose a standardized difference of 0.25 rather than 0.1 for the final analysis, as suggested by some statisticians [25]. We used a multistep approach to evaluate the association between TXA administration and in-hospital mortality across different trauma phenotypes. This analysis involved secondary use of existing data; therefore, the exact sample size was not calculated.

### Handling of missing data and clustering

In the naïve dataset, multicollinearity issues among the variables used for clustering were assessed using variance inflation factors with a threshold of < 2. The 14 variables used for clustering to identify trauma phenotypes included information obtained during the initial assessment of trauma care: patient background (age, sex, and number of underlying diseases), vital signs (respiratory rate, heart rate, systolic blood pressure, Glasgow Coma Scale score, and body temperature), and AIS codes (six regions) [18]. Clustering requires that there are no deficiencies in these 14 variables. Therefore, the missing values of the 14 variables used for clustering were examined, and multiple imputations using all 14 variables were made to the variables with the missing values. Missing data in the naïve dataset were assumed to be missing at random, and multiple imputation was used to handle the missing data. The multiple imputation by chained equations package was used to construct multiple regression models, including variables potentially related to missing data, to impute the missing data [26]. After 15 iterations, 30 imputed datasets were created, and the convergence of the imputed values was verified using convergence plots. The validity of the imputation algorithm was confirmed by overlaying the distribution of the imputed values on the originally observed values and examining the density functions. Eight trauma phenotypes were obtained through clustering using derivation cohort data (JTDB data between January 2013 and June 2015) from a previous study [18]. The current study adhered to this classification. Therefore, using the same variables as in the previous study, new data were mapped to the trauma phenotypes obtained in the previous study. Euclidean distances were calculated for imputed and standardised continuous and categorical variables to estimate the similarity between patients. The nearest-neighbour method (*k* = 5) was then used to assign the imputed data to the existing clusters. As a result, D-1 in the previous study was mapped to Trauma phenotype 1 in this study, D-2 in the previous study to Trauma phenotype 2 in this study, and so on. The distribution of in-hospital mortality and covariates in each cluster was visualised using complex heatmaps. Furthermore, to grasp the characteristics of each trauma phenotype, a summary of clinical features was compiled.

### Inverse probability treatment weighting

Given the retrospective nature of this study, we assumed a group imbalance based on baseline covariates. Inverse probability treatment weighting (IPTW) using propensity score methods was used to address this imbalance and adjust for the confounding factors. The IPTW uses propensity scores to adjust for potential confounders while maintaining the sample size [27]. A logistic regression model was used to estimate the propensity scores with the following independent variables: age, sex, number of comorbidities, vital signs upon admission, and trauma severity (AIS codes of each of the six body regions). The primary outcomes were compared using a logistic regression model to calculate the odds ratio (OR) of in-hospital mortality based on TXA administration within each cluster using IPTW propensity scores. This analysis controlled for biases between the TXA-administered and non-administered groups, assessing the impact of TXA administration on in-hospital mortality, with trauma phenotypes as subgroups (predictive approach to heterogeneous treatment effects). Furthermore, a logistic regression model with in-hospital mortality and TXA administration as the dependent and sole independent variables (without weighting), respectively, was used for crude analysis to verify the adjustment of confounders using IPTW.

### Integration of results and visualization

The data obtained through these methods were analysed using descriptive and inferential statistics. Descriptive statistics were used to examine the distributions of categorical and numerical data. Multiple imputed datasets, integrated using Rubin’s rule [28], were used for inferential statistics to calculate point estimates within a 95% confidence interval. Stouffer’s method was used in the multiple imputation analysis to calculate the combined *p*-values for each cluster. Regarding multiple comparisons, after aggregating the results for each cluster, the Benjamini–Hochberg procedure was applied to the *p*-values across all clusters to calculate the adjusted *p*-values. Forest plots were used to visualise the analysis results regarding the relative effects of TXA and their statistical significance.

### Sensitivity analysis

A sensitivity analysis was conducted to test the robustness of the results using generalised linear mixed models to account for treatment heterogeneity among facilities. This approach addresses concerns regarding differences in the characteristics of patients with trauma admitted to each facility and variations in treatment approaches, including TXA administration. The fixed-effect variables were the same as those used to estimate the propensity scores, and the hospital-specific identifiers were random-effect variables. Additionally, to verify the validity of the missing data mechanism assumption, an analysis was conducted on complete cases using IPTW [29]. Additionally, to assess the effect of the timing of TXA administration, a subgroup analysis was conducted on patients admitted within 2 h of injury. In the statistical analysis of this study, multiple statistical methods were used to control for confounding factors and evaluate the effect of TXA on patients with blunt trauma. Logistic regression models, IPTW with propensity scores, and generalised linear mixed models were used to ensure the robustness of the results obtained in this study. All statistical results were point estimates within 95% confidence intervals (CIs), and the threshold for statistical significance was set at a *p*-value of < 0.05 based on two-sided tests.

All statistical analyses were performed using “R 4.3.1” for statistical computing (R Foundation for Statistical Computing, Vienna, Austria, https:// www.r-project. ORG/), using several add-on packages. This study followed the STROBE guidelines [30].

## Results

### Study population

**Fig. 1:**
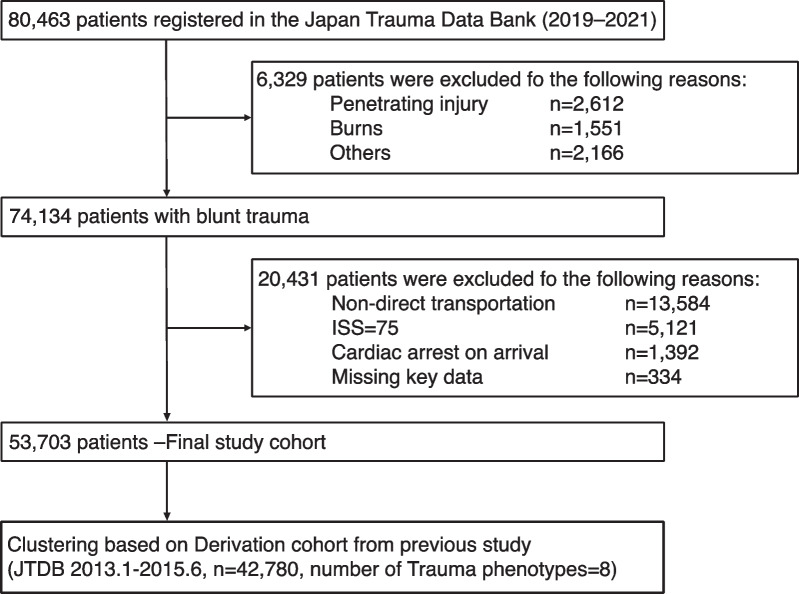
Flow chart of patient selection

Overall, 80,463 patients with trauma were registered between 2019 and 2021 in the JTDB. Of these, 74,134 patients with blunt trauma were included in this study. After excluding 20,431 patients who met the exclusion criteria, data of 53,703 were analysed (Fig. 1). Of these, 8046 (15.0%) and 45,657 (85.0%) patients constituted the TXA administration and non-administration groups, respectively. Table 1 presents the baseline characteristics of both groups before and after IPTW adjustment (left side: unweighted cohort). The overall mean age of the study population was 62.2 years, with an average ISS of 14.5, and the in-hospital mortality rate was 6.1% (3286 out of 53,703 cases). In the unweighted cohort, patients in the TXA group were slightly younger (mean age, 62.6 vs. 60.0 years, ASMD = 0.111) and constituted higher proportions of male patients (59.5% vs. 68.5%, ASMD = 0.190) and those with more severe trauma (mean ISS, 13.2 vs. 21.7, ASMD = 0.886).

**Table 1:** Patient characteristics in the weighted and unweighted cohorts in the study group

Variable	Unweighted cohort			Weighted cohort		
	TXA non-treated (*n* = 45,657)	TXA treated (*n* = 8046)	ASMD	TXA non-treated (*n* = 54,271)	TXA treated (*n* = 48,423)	ASMD
*Demographics*						
Age, mean (years)	62.6	60.0	0.111	62.0	60.0	0.090
Male sex (%)	59.5	68.5	0.190	61.1	65.9	0.100
Number of comorbidities	0.6	0.5	0.092	0.57	0.49	0.078
*Vital signs*						
Respiratory rate, mean (/min)	20	22	0.242	21	21	0.056
Heart rate, mean (bpm)	85	90	0.247	86	87	0.022
Systolic blood pressure, mean (mmHg)	142	136	0.166	141	138	0.074
Glasgow Coma Scale score, mean	13.7	12	0.516	13	13	0.072
Body temperature, mean (℃)	36.6	36.3	0.237	36.5	36.4	0.068
*Trauma severit*						
Head & Cervical AIS, mean	1.3	2.6	0.674	1.6	1.8	0.117
Face AIS, mean	0.1	0.3	0.256	0.2	0.2	0.035
Chest AIS, mean	0.8	1.4	0.381	0.96	1.1	0.089
Abdomen AIS, mean	0.4	0.7	0.343	0.4	0.5	0.062
Extremities AIS, mean	1.5	1.3	0.086	1.5	1.3	0.077
External AIS, mean	0.3	0.4	0.198	0.3	0.3	0.062
ISS, mean	13.2	21.7	0.886	14.9	16.2	0.133

*TXA* Tranexamic acid, *ASMD* Absolute standardised mean difference, *AIS* abbreviated injury scale, *ISS* Injury severity score

### Missing data: multiple imputation

The vital sign data had missing values (Additional file 1: Table S1), which were distributed randomly (Additional file 1: Fig. S1). Convergence was generally observed for each variable following multiple imputations (Additional file 1: Fig. S2), and the distribution of variables after imputation revealed no significant differences from the original data (Additional file 1: Fig. S3).

### IPTW

In the weighted cohort, the ASMDs for key variables such as age, sex, number of comorbidities, vital signs, and trauma severity were < 0.25, indicating a balanced group (Table 1, right side). These results confirmed that the differences in patient characteristics between the TXA administration and non-administration groups were significantly reduced through IPTW adjustment, making the groups comparable.

### Clustering

**Fig. 2:**
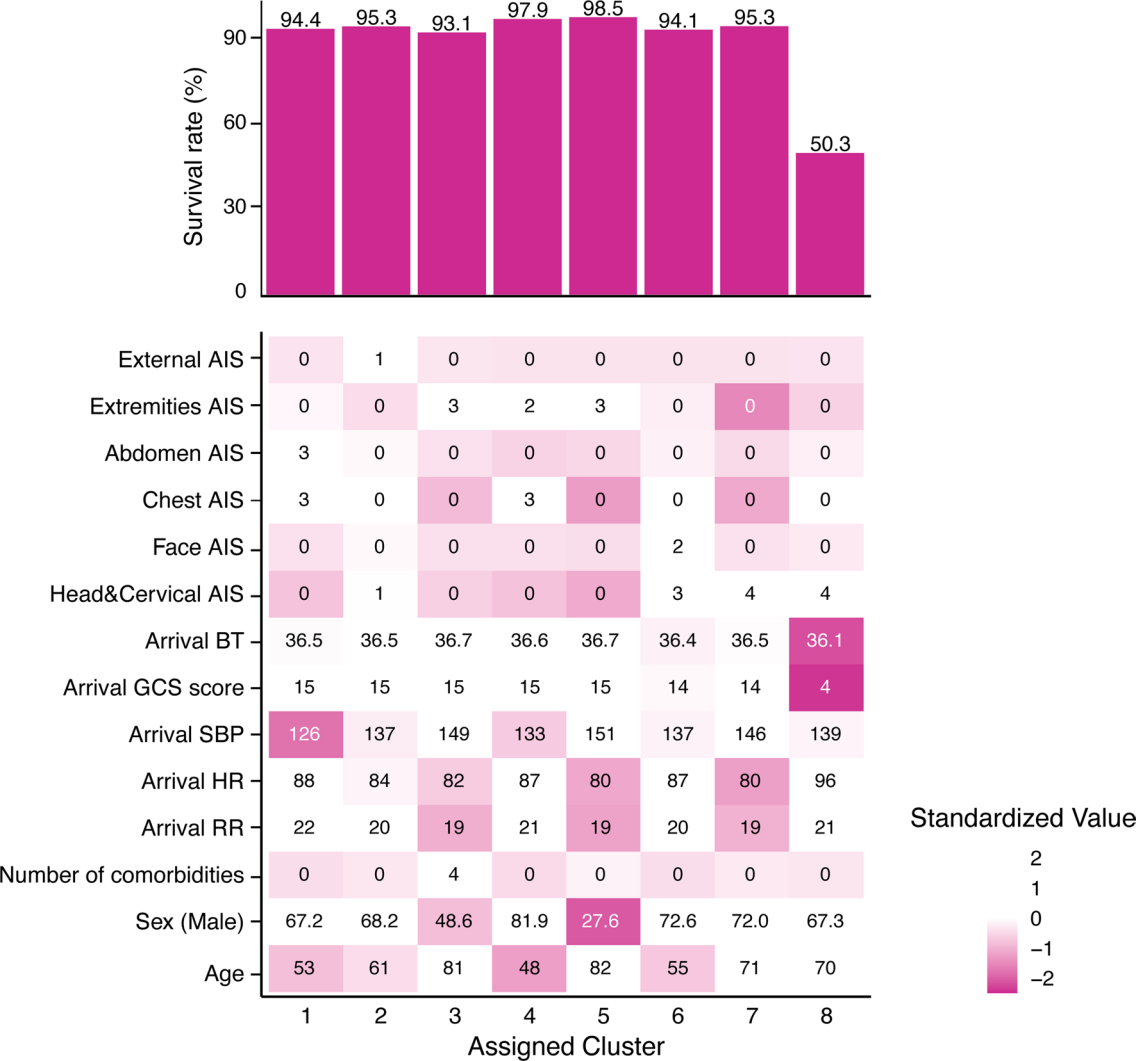
Complex heatmap with the distribution of survival rates and variables for each trauma phenotype. Complex heat map presents the distribution of survival rates and variables for each trauma phenotype using multiple imputation data. Heatmap reveals the distribution of survival rates and variables for each clinical phenotype. The upper panel presents the survival rate for each clinical phenotype in the bar graphs. The heat map in the bottom panel presents each variable (standardised and coloured). The number of cells is presented as the median (sex is presented as the percentage of males). *AIS* Abbreviated injury scale, *BT* Body temperature, *GCS* Glasgow Coma Scale, *SBP* Systolic blood pressure, *HR* Heart rate, *RR* Respiratory rate

**Fig. 3:**
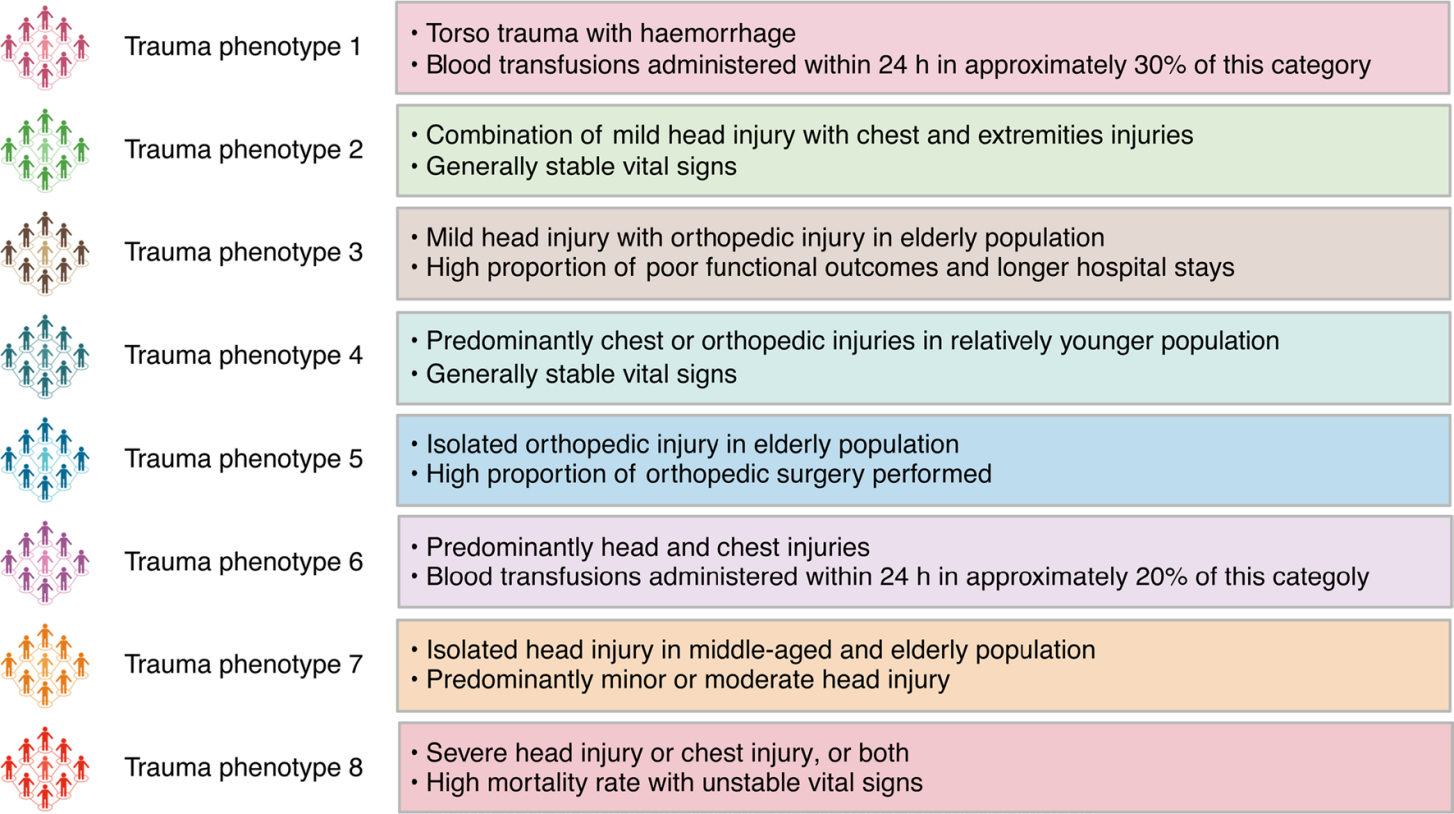
Summary of clinical features in each trauma phenotype

Examination of the variance inflation factors for the variables used in clustering revealed that none exceeded 0.2, indicating that multicollinearity issues were addressed (Additional file 1: Fig. S4). Table 2 presents the patient number and baseline characteristics of the eight trauma phenotypes (one dataset from multiple imputed datasets is shown for reference). Figure 2 illustrates the survival rates and distributions of various variables for each assigned trauma phenotype. A summary of clinical features of each trauma phenotype is shown in Fig. 3. Trauma phenotype 8 with a high mortality rate was characterised by severe head injuries, a tendency towards lower body temperatures, and a mortality rate of 50.2%, exhibiting characteristics equivalent to those of high-mortality phenotypes in previous studies [18].

**Table 2:** Characteristics of patients with each trauma phenotype

	Trauma phenotype 1	Trauma phenotype 2	Trauma phenotype 3	Trauma phenotype 4	Trauma phenotype 5	Trauma phenotype 6	Trauma phenotype 7	Trauma phenotype 8	Overall
Number of Patients	4791	14,579	2025	7,221	12,000	1,920	8,399	2,768	53,703
Age, years, median [IQR]	53 [33, 71]	61 [37, 77]	81 [73, 87]	48 [26, 67]	82 [72, 88]	55 [35, 72]	71 [56, 81]	70 [50, 81]	69 [47, 81]
Male sex, No. (%)	3,229 (67.4)	9,945 (68.2)	985 (48.6)	5,914 (81.9)	3,305 (27.5)	1,398 (72.8)	6,042 (71.9)	1,852 (66.9)	32,670 (60.8)
Number of comorbidities, median [IQR]	0 [0, 0]	0 [0, 1]	4 [3, 5]	0 [0, 0]	0 [0, 1]	0 [0, 0]	0 [0, 1]	0 [0, 1]	0 [0, 1]
Respiratory rate (/min), median [IQR]	22 [18, 27]	20 [17, 24]	19 [16, 22]	21 [18, 25]	18 [16, 21]	20 [18, 24]	19 [16, 22]	20 [17, 25]	20 [17, 24]
Heart rate (bpm), median [IQR]	87 [75, 102]	84 [73, 98]	82 [72, 94]	87 [75, 100]	80 [70, 91]	87 [75, 102]	80 [69, 91]	94 [76, 114]	83 [72, 97]
Systolic blood pressure (mmHg), median [IQR]	126 [106, 145]	137 [118, 158]	149 [128, 169]	133 [117, 151]	151 [131, 170]	137 [120, 157]	146 [126, 168]	140 [110, 170]	140 [120, 162]
Systolic blood pressure (mmHg) ≤ 90, No. (%)	655 (13.7)	881 (6.0)	57 (2.8)	359 (5.0)	221 (1.8)	104 (5.4)	274 (3.3)	440 (15.9)	2,991 (5.6)
Glasgow Coma Scale score, median [IQR]	15 [14, 15]	15 [14, 15]	15 [14, 15]	15 [14, 15]	15 [15]	14 [11, 15]	15 [13, 15]	3 [3, 6]	15 [14, 15]
*Glasgow Coma Scale category*									
13–15, No. (%)	4,192 (87.5)	12,243 (84.0)	1,878 (92.7)	6,782 (93.9)	11,725 (97.7)	1,344 (70.0)	7,127 (84.9)	12 (0.4)	45,303 (84.4)
9–12, No. (%)	337 (7.0)	1,028 (7.1)	94 (4.6)	346 (4.8)	250 (2.1)	244 (12.7)	1,062 (12.6)	58 (2.1)	3,419 (6.4)
3–8, No. (%)	262 (5.5)	1,308 (9.0)	53 (2.6)	93 (1.3)	25 (0.2)	332 (17.3)	210 (2.5)	2,698 (97.5)	4,981 (9.3)
Body temperature (℃), median [IQR]	36.5 [36.0, 36.9]	36.5 [36.1, 36.9]	36.7 [36.3, 37.1]	36.6 [36.2, 37.0]	36.7 [36.4, 37.1]	36.4 [36.0, 36.8]	36.5 [36.1, 36.8]	36.2 [35.6, 36.6]	36.6 [36.1, 36.9]
Lactate on arrival (mmol/L), median [IQR]	2.40 [1.55, 3.66]	2.00 [1.38, 3.00]	1.51 [1.00, 2.22]	2.03 [1.40, 3.00]	1.55 [1.09, 2.23]	2.30 [1.55, 3.33]	1.79 [1.22, 2.66]	3.00 [1.80, 5.90]	1.96 [1.30, 3.00]
*Injured body regions of AIS* > 2									
Head & Cervical, No. (%)	640 (13.4)	6,026 (41.3)	429 (21.2)	895 (12.4)	330 (2.8)	1,231 (64.1)	7,791 (92.8)	2,504 (90.5)	19,846 (37.0)
Face, No. (%)	1 (0.0)	67 (0.5)	0 (0.0)	0 (0.0)	0 (0.0)	118 (6.1)	0 (0.0)	4 (0.1)	190 (0.4)
Chest, No. (%)	2,436 (50.8)	4,272 (29.3)	244 (12.0)	4,092 (56.7)	530 (4.4)	650 (33.9)	504 (6.0)	990 (35.8)	13,718 (25.5)
Abdomen, No. (%)	2,442 (51.0)	910 (6.2)	43 (2.1)	0 (0.0)	19 (0.2)	73 (3.8)	28 (0.3)	116 (4.2)	3,631 (6.8)
Extremities, No. (%)	1,049 (21.9)	2,567 (17.6)	1,245 (61.5)	1,957 (27.1)	9,954 (83.0)	364 (19.0)	133 (1.6)	442 (16.0)	17,711 (33.0)
External, No. (%)	0 (0.0)	110 (0.8)	0 (0.0)	0 (0.0)	0 (0.0)	0 (0.0)	0 (0.0)	0 (0.0)	110 (0.2)
Multiple body parts with AIS > 2 injuries, No. (%)	1,912 (39.9)	2,717 (18.6)	108 (5.3)	1,110 (15.4)	310 (2.6)	645 (33.6)	600 (7.1)	1,018 (36.8)	8,420 (15.7)
RTS, median [IQR]	7.84 [7.55, 7.84]	7.84 [7.84, 7.84]	7.84 [7.84, 7.84]	7.84 [7.84, 7.84]	7.84 [7.84, 7.84]	7.84 [6.90, 7.84]	7.84 [7.84, 7.84]	5.03 [4.09, 5.97]	7.84 [7.84, 7.84]
ISS, median [IQR]	17 [9, 25]	14 [9, 21]	9 [9]	9 [9, 16]	9 [9]	20 [13, 29]	16 [9, 18]	25 [17, 32]	10 [9, 18]
TRISS ps, median [IQR]	0.97 [0.91, 0.99]	0.96 [0.93, 0.99]	0.97 [0.96, 0.97]	0.99 [0.96, 0.99]	0.97 [0.97, 0.97]	0.94 [0.85, 0.98]	0.94 [0.92, 0.97]	0.45 [0.27, 0.72]	0.97 [0.93, 0.98]
Transfusion within 24 h, No. (%)	1,462 (31.5)	2,043 (14.6)	213 (10.8)	770 (11.2)	962 (8.3)	430 (23.1)	566 (7.0)	1,139 (43.2)	14.7 (7,585)
pRBC within 24 h, units, median [IQR]	6 [4, 14]	4 [2, 8]	2 [2, 4]	4 [2, 10]	2 [2, 4]	6 [4, 10]	4 [0, 6]	6 [4, 12]	4 [2, 10]
FFP within 24 h, units, median [IQR]	8 [4, 16]	6 [2, 12]	2 [0, 6]	6 [4, 12]	2 [0, 6]	8 [4, 14]	4 [2, 10]	8 [4, 16]	6 [4, 12]
PC within 24 h, units, median [IQR]	0 [0, 20]	0 [0, 10]	0 [0, 0]	0 [0, 10]	0 [0, 0]	0 [0, 10]	0 [0, 10]	0 [0, 20]	0 [0, 10]
Administration of TXA, No. (%)	1,081 (22.6)	2,796 (19.2)	139 (6.9)	776 (10.7)	397 (3.3)	476 (24.8)	1,488 (17.7)	893 (32.3)	8,046 (15.0)
Vasopressor, No. (%)	294 (6.1)	489 (3.4)	10 (0.5)	121 (1.7)	35 (0.3)	77 (4.0)	98 (1.2)	501 (18.1)	1,625 (3.0)
Thoracic drainage, No. (%)	616 (12.9)	804 (5.5)	38 (1.9)	892 (12.4)	55 (0.5)	146 (7.6)	64 (0.8)	339 (12.2)	2,954 (5.5)
REBOA, No. (%)	524 (10.9)	722 (5.0)	28 (1.4)	204 (2.8)	79 (0.7)	177 (9.2)	153 (1.8)	505 (18.2)	2,392 (4.5)
Head surgery, No. (%)	42 (0.9)	364 (2.5)	49 (2.4)	53 (0.7)	16 (0.1)	91 (4.7)	577 (6.9)	609 (22.0)	1,801 (3.4)
Cervical surgery, No. (%)	9 (0.2)	63 (0.4)	2 (0.1)	13 (0.2)	1 (0.0)	11 (0.6)	59 (0.7)	26 (0.9)	184 (0.3)
Chest surgery, No. (%)	144 (3.0)	155 (1.1)	6 (0.3)	123 (1.7)	11 (0.1)	27 (1.4)	7 (0.1)	102 (3.7)	575 (1.1)
Abdominal surgery, No. (%)	983 (20.5)	459 (3.1)	15 (0.7)	30 (0.4)	27 (0.2)	46 (2.4)	17 (0.2)	85 (3.1)	1,662 (3.1)
Orthopedic surgery, No. (%)	1,486 (31.0)	3,070 (21.1)	1,035 (51.1)	2,485 (34.4)	8,362 (69.7)	430 (22.4)	773 (9.2)	231 (8.3)	17,872 (33.3)
Orthopedic IVR, No. (%)	263 (5.5)	343 (2.4)	17 (0.8)	117 (1.6)	141 (1.2)	56 (2.9)	25 (0.3)	90 (3.3)	1,052 (2.0)
*Glasgow outcome scale, No*. (%)									
Dead	270 (5.6)	691 (4.7)	138 (6.8)	151 (2.1)	173 (1.4)	113 (5.9)	392 (4.7)	1,358 (49.1)	3,286 (6.1)
Vegetative state	11 (0.2)	77 (0.5)	15 (0.7)	5 (0.1)	18 (0.2)	18 (0.9)	47 (0.6)	102 (3.7)	293 (0.5)
Severely disabled	483 (10.1)	1,538 (10.5)	467 (23.1)	483 (6.7)	2,592 (21.6)	199 (10.4)	1,171 (13.9)	369 (13.3)	7,302 (13.6)
Moderately disabled	621 (13.0)	1,764 (12.1)	296 (14.6)	863 (12.0)	1,641 (13.7)	244 (12.7)	1,023 (12.2)	186 (6.7)	6,638 (12.4)
Good recovery	1,416 (29.6)	4,937 (33.8)	339 (16.7)	2,372 (32.8)	2,035 (17.0)	610 (31.8)	2,351 (28.0)	169 (6.1)	14,229 (26.5)
Unknown	1,990 (41.5)	5,572 (38.2)	770 (38.0)	3,347 (46.4)	5,541(46.2)	736 (38.3)	3,415 (40.7)	584 (21.1)	21,955 (40.9)
Length of hospital stay, days, median [IQR]	20 [10, 36]	11 [2, 26]	21 [13, 32]	12 [4, 26]	20 [13, 30]	19 [7, 35]	14 [5, 28]	15 [1, 41]	16 [6, 29]
Survive, No. (%)	4,521 (94.4)	13,888 (95.3)	1,887 (93.2)	7,070 (97.9)	11,827 (98.6)	1,807 (94.1)	8,007 (95.3)	1,410 (50.9)	50,417 (93.9)

This table shows the baseline characteristics for each trauma phenotype (one dataset from multiple imputed datasets is shown for reference). The quantity of transfusion products represents the total amount of blood products used during hospitalisation and is presented according to the dosing standards for blood products in Japan. Head surgery includes craniotomy, trephination, and placement of intracranial pressure (ICP) sensors. Cervical surgery encompasses surgical treatments, including interventional radiology (IVR). Chest surgery comprises thoracotomy, video-assisted thoracic surgery (VATS), and IVR but does not include resuscitative thoracotomy. Abdominal surgery involves laparotomy, laparoscopic surgery, and IVR. Orthopedic surgery includes open reduction, amputation, and external fixation procedures. Orthopedic IVR covers IVR for pelvic, limb, and spinal injuries

*Abbreviations*: IQR, interquartile range; AIS, abbreviated injury scale; RTS, revised trauma score; ISS, injury severity score; TRISS, trauma and injury severity score probability of survival; pRBC, packed red blood cell; FFP, fresh frozen plasma; PC, platelet concentrate; TXA, tranexamic acid; REBOA, resuscitative endovascular balloon occlusion of the aorta; IVR, interventional radiology

### Association between TXA administration and in-hospital mortality based on the trauma phenotype

**Fig. 4:**
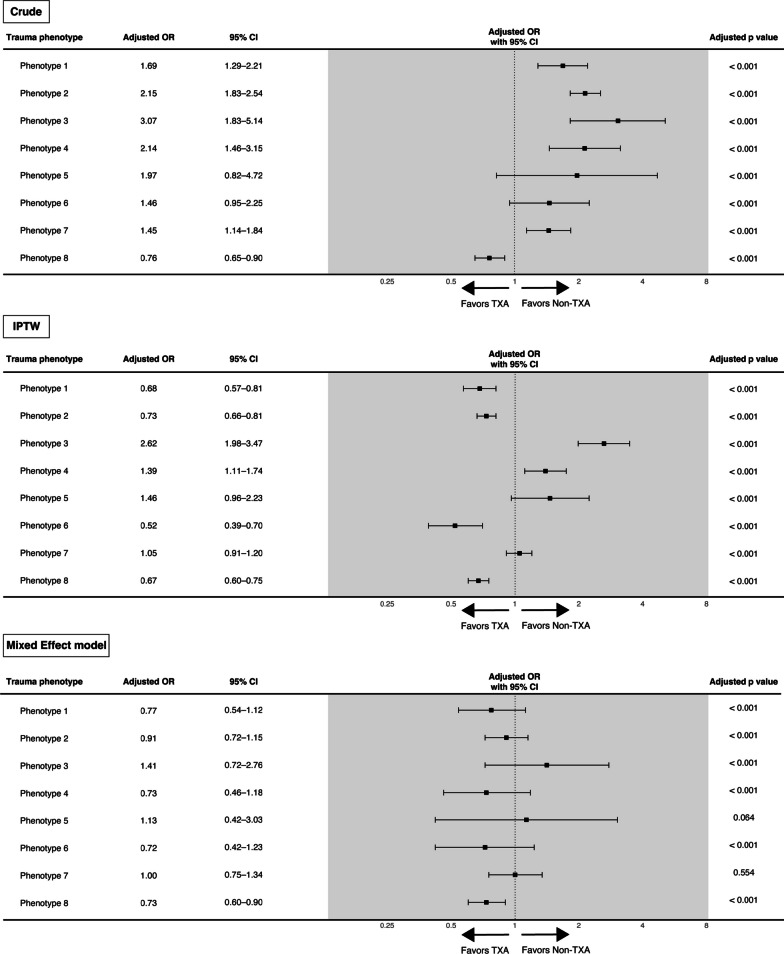
Association between TXA and in-hospital mortality based on the trauma phenotype. Three analytical methods were used to evaluate the in-hospital mortality rate associated with TXA for each trauma phenotype. Crude analysis: using a logistic regression model with in-hospital mortality as the dependent variable and TXA administration as the sole independent variable (without weighting), IPTW: primary analysis, mixed-effect model: analysis incorporating medical institutions as random effects. *OR* Odds ratio, *CI* Confidence interval, *TXA* Tranexamic acid, *IPTW* Inverse probability treatment weighting

Figure 4 presents the ORs for in-hospital mortality associated with TXA administration, adjusted using a logistic regression model. In the primary analysis using IPTW, the odds of mortality associated with TXA administration were significantly lower in trauma phenotypes 1, 2, 6, and 8 and significantly higher in 3 and 4.

### Sensitivity analysis

In the generalised linear mixed models with facility as a random effect, only trauma phenotype 8 had significantly lower odds of mortality associated with TXA administration (OR 0.71, 95% CI 0.58–0.87, Fig. 3). In the analysis using IPTW for complete cases, a similar trend was observed as in the analysis with multiple imputations (Additional file 1: Fig. S5). Additionally, among patients with known time from injury to hospital arrival (74.0%, 39,739/53,703), 92.8% (5,157/5,559) in the TXA administration group and 82.3% (28,118/34,180) in the non-administration group arrived at the hospital within 120 min after injury (Additional file 1: Fig. S6). In the subgroup analysis of patients with complete information and with a clear time from injury to hospital arrival, the TXA administration group with trauma phenotypes 1, 2, 6, and 8 had significantly lower odds of mortality (Additional file 1: Fig. S7).

## Discussion

This study used JTDB data to investigate the association between TXA administration and the survival outcome based on trauma phenotypes. Consequently, subgroups were identified within specific trauma phenotypes that may be associated with TXA administration and survival outcomes. These results were supported by sensitivity analyses and contribute to our current understanding of TXA efficacy.

This study has two significant clinical implications. First, it identified subgroups of all patients with blunt trauma for whom TXA administration was likely beneficial and those for whom it was not advisable. Evidence exists for TXA administration in patients at risk of significant haemorrhagic and those with mild-to-moderate head injuries; nonetheless, the truly effective target population for TXA has not been sufficiently clarified. A strength of this study is that it encompassed all patients with blunt trauma, including those with minor injuries, and comprehensively examined the association between TXA administration and the survival outcome based on the trauma phenotype. Additionally, the use of an integrated model that incorporates factors, such as comorbidities, vital signs upon admission, and the type and extent of organ damage, allows for an analysis that considers the heterogeneity of patients with trauma and adds novelty to the study. Meanwhile, studies examining the treatment effect of TXA by baseline risk based on RCTs showed no heterogeneity [31, 32]. This result is at variance with the hypothesis of this study. We aimed to identify subgroups—defined by trauma phenotypes developed in our previous study—that might particularly benefit from TXA, thereby reducing intragroup heterogeneity. Although our study is not a secondary analysis of RCT data and may not be as robust, it serves as an important step in hypothesis generation for future studies. Clinically, the clusters in which TXA administration resulted in reduced mortality rates (trauma phenotypes 1, 2, 6, and 8) exhibited higher median ISSs and a tendency towards greater trauma severity. The PATCH trauma trial included patients with severe multisite injuries and reported reduced mortality rates at 24 h and 1 month after injury in a secondary analysis, consistent with the results for severe trauma phenotypes in this study [9]. In our previous study, proteomic analyses of the molecular pathology of high-mortality phenotypes (trauma phenotype 8 in this study) demonstrated the involvement of coagulation disorders (hyperfibrinolysis) [18]. These findings explain the effectiveness of TXA in this study from a molecular pathology perspective. However, for trauma phenotypes 3 and 4, TXA administration was associated with increased in-hospital mortality rates. Trauma phenotype 3 is characterised by orthopaedic injuries in elderly population, trauma phenotype 4 is primarily seen in younger patients with chest or orthopedic injury, and both are associated with lower trauma severity levels. Additionally, the lower proportion of patients who were administered TXA in these clusters, owing to the minor nature of the injuries, may have amplified the effect of the few mortality cases. Nevertheless, this study represents the first step toward the practice of personalized medicine in trauma care, predicting the efficacy of therapeutic interventions in a highly heterogeneous population. Although accumulating many cases in prospective studies is challenging, assessing the association between TXA administration and the survival outcome using statistical methods is a strength. Validating these results in future prospective studies could lead to more robust and optimised TXA administration.

Second, trauma phenotypes can be identified based on the information available at the early stages of clinical assessment. Early administration of TXA within 3 h post-injury is recommended; therefore, identifying ideal candidates for early administration is crucial. Early intervention is the key to trauma treatment strategies [33]; therefore, efficiently identifying suitable candidates for treatment at a treatable stage is essential. Identifying trauma phenotypes requires the AIS, which necessitates various imaging tests for determination. Spending time on identifying trauma phenotypes risks missing the golden hour for administering TXA, especially in patients whose benefits from TXA have already been established in previous large-scale studies [5, 7], only to base administration on these identifications. For rapidly identifying trauma phenotypes, applications to detect the trauma phenotype and the revision of clinical workflows, such as integrating computed tomography (CT) scans into trauma resuscitation [34, 35], which allow for early CT imaging and, thus, swift identification of trauma phenotypes following imaging, have been developed. While there are many challenges to implementing TXA administration based on trauma phenotypes, this study serves as a hypothesis-generating investigation. Through future validation, it may enable more appropriate targeting of the patient population for whom TXA administration is desirable.

By evaluating the association between TXA administration and the survival outcome based on trauma phenotypes obtained using clinical information in heterogeneous diseases can reduce the variability in treatment effects, potentially leading to more efficient clinical research implementation (predictive enrichment) [15, 36]. Moreover, in several research fields, treatment efficacy has been validated through pre-subgrouping (phenotyping) based on machine learning, thus facilitating more efficient clinical trials [37–39]. Similar to other studies, future research should elucidate and clinically interpret the molecular pathological features of each trauma phenotype, using trauma phenotypes as an enrichment approach.

This study has some limitations. First, as this study was retrospective in nature, it inherently possessed specific limitations such as the inability to establish causation, potential selection bias in the choice of participants, regression dilution bias, and unmeasured confounding variables that may influence the outcomes. Notably, the database used in this study did not include the timing of TXA administration. Additionally, the time of injury was unknown in 26.0% of the analysed data; thus, the duration from injury to hospital admission was unclear. Early administration of TXA within 3 h after injury is recommended, and delays in administration are potentially harmful [6]. In trauma phenotypes 3, 4, and 5, administration of TXA was associated with an increase in in-hospital mortality, suggesting a possible delay in TXA administration. In the subgroup analysis of cases with known injury times, 92.8% of the patients in the TXA administration group arrived at the hospital within 120 min post-injury, and the sensitivity analysis for these cases yielded results consistent with those of the primary analysis.

Second, the TXA administration protocol was unclear. The Japan Advanced Trauma Evaluation and Care guidelines [40] describe the use of TXA within 3 h post-injury for traumas with a high bleeding risk or mild to moderate traumatic brain injury, as an adjunctive haemostatic therapy, based on the results of two large RCTs [5, 7]. Medical institutions generally follow these guidelines; nonetheless, the degree of adherence varies. Consequently, the study conducted sensitivity analyses considering the facility as having a mixed effect, revealing that TXA administration was favourable in the most severe trauma clusters even after accounting for facility-related influences. Third, only point-of-admission data were considered. This limitation implies that temporal changes in the patient’s condition were not accounted for. This is a significant limitation in assessing the effects of TXA on evolving clinical conditions, suggesting that future research should analyse longitudinal data. Fourth, the exclusion criteria were slightly different from those in the previous study [18]. As the JTDB was modified in the period between the previous and present studies, obtaining information related to pregnancy was no longer possible. However, pregnant women represented only approximately 0.1% of the excluded patients in the previous study; assuming that the frequency was similar in the present study, the impact on the results was likely to be limited. Fifth, our study’s data, sourced solely from Japanese patients, have a limitation in external validity. Our cohort’s mean age was higher and the trauma severity lower than those of other cohorts [41], with physiological parameters not reflecting severe conditions. This discrepancy might stem from the specific criteria for registry enrolment and Japan’s demographic profile, which is characterized by a high aging population among developed nations [42]. Such demographic and clinical features raise concerns about the applicability of our findings to other populations. Consequently, external validation with other trauma cohorts is essential to confirm the robustness of our results. Sixth, despite weighting by IPTW, the ASMD for head injury and ISS exceeded 0.1, which usually indicates an imbalance, and this could be considered a limitation of this study. Meanwhile, based on a study that the ASMD must be smaller than 0.25 for the regression adjustment to be reliable [25], we judge this imbalance to be within acceptable limits.

## Conclusions

In summary, this retrospective analysis of a national cohort of patients with blunt trauma evaluated the association between TXA administration and the survival outcome based on the trauma phenotype identified using available clinical information at the early stages of trauma care. This study indicates that TXA administration has the potential to significantly reduce or increase in-hospital mortality rates in patients with certain trauma phenotypes. Our findings suggest variability in the effectiveness of TXA among patients with trauma; further research with an independent dataset with higher external validity and prospective data is needed to confirm our findings. This study could be an offshoot towards the implementation of enrichment strategies in trauma care.

### Supplementary Information

**Additional file 1:** Supplemental Digital Contents.

## Data Availability

The datasets used in the current study are available from the authors upon reasonable request.

## Abbreviations

TIC: Trauma-induced coagulopathy
TXA: Tranexamic acid
RCT: Randomised controlled trial
ARDS: Acute respiratory distress syndrome
AIS: Abbreviated injury scale
ISS: Injury severity score
JTDB: Japan Trauma Data Bank
ASMD: Absolute standardised mean difference
IPTW: Inverse probability treatment weighting
OR: Odds ratio
CI: Confidence interval
