# Supplementary material for: Association between tranexamic acid administration and mortality based on the trauma phenotype: a retrospective analysis of a nationwide trauma registry in Japan
Source: Crit Care. 2024 Mar 19;28:89. doi: 10.1186/s13054-024-04871-w (PMC10953216; doi:10.1186/s13054-024-04871-w)
Supplement: Supplementary file 1 — Additional file 1: Supplemental Digital Contents. [file 13054_2024_4871_MOESM1_ESM.docx]

**Supplemental Digital Contents**

**Association between tranexamic acid administration and mortality based on the trauma phenotype: a retrospective analysis of a nationwide trauma registry in Japan**

***Database***

The Japan Trauma Data Bank (JTDB), established in 2003, is a comprehensive trauma registry encompassing data from 303 hospitals as of March 2021. Initiated by the Japanese Association for the Surgery of Trauma (Trauma Registry Committee) and the Japanese Association for Acute Medicine (Committee for Clinical Care Evaluation), the JTDB aims to enhance the quality of trauma care across Japan. The data, updated continuously, are shared online and stored on the data server of the Association for Japan Trauma Care and Research. The JTDB primarily records patients from tertiary care and emergency centers, suspected of sustaining injuries with an Abbreviated Injury Scale score of 3 or higher, and tracks their progress until hospital discharge or death.

**Supplementary Tables**

**Supplemental Table 1. Number of missing data for each variable**

| **Variable** | **TXA non-treated** | **TXA treated** | **All Patients** |
| --- | --- | --- | --- |
| Number of Patients | 45,657 | 8,046 | 53,703 |
| Age, years, median [IQR] | 69 [47-82] | 66 [45-78} | 69 [47-81] |
| Unknown | 0 (0) | 0 (0) | 0 (0) |
| Male gender No. (%) | 27,166 (59.5) | 5,512 (68.5) | 32,678 (60.8) |
| Unknown | 0 (0) | 0 (0) | 0 (0) |
| Systolic blood pressure (mmHg) ,median [IQR] | 141 [122-162] | 136 [113-160] | 140 [120-162] |
| Unknown | 780 (1.7) | 66 (0.8) | 846 (1.6) |
| Respiratory rate (/min), median [IQR] | 20 [17-23] | 20 [18-25] | 20 [17-24] |
| Unknown | 3,036 (6.6) | 339 (4.2) | 3,375 (6.3) |
| Heart rate (bpm), median [IQR] | 83 [72-95] | 87 [74-103] | 83[72-96] |
| Unknown | 979 (2.1) | 75 (0.9) | 1,054 (2) |
| Body temperature (℃), median [IQR] | 36.6 [36.2-37] | 36.4 [36-36.8] | 36.6 [36.1-36.9] |
| Unknown | 2,706 (5.9) | 604 (7.5) | 3,310 (6.2) |
| Glasgow Coma Scale score, median [IQR] | 15 [14-15] | 14 [10-15] | 15 [14-15] |
| Unknown | 1,733 (3.8) | 144 (1.8) | 1,877 (3.5) |
| Number of comorbidities, median [IQR] | 0 [0-1] | 0 [0-1] | 0 [0-1] |
| Unknown | 0 (0) | 0 (0) | 0 (0) |
| Head & Cervical AIS, median [IQR] | 0 [0-3] | 3 [0-4] | 0 [0-3] |
| Unknown | 0 (0) | 0 (0) | 0 (0) |
| Face AIS, median [IQR] | 0 [0-0] | 0 [0-0] | 0 [0-0] |
| Unknown | 0 (0) | 0 (0) | 0 (0) |
| Chest AIS, median [IQR] | 0 [0-2] | 0 [0-3] | 0 [0-3] |
| Unknown | 0 (0) | 0 (0) | 0 (0) |
| Abdomen AIS, median [IQR] | 0 [0-0] | 0 [0-2] | 0 [0-0] |
| Unknown | 0 (0) | 0 (0) | 0 (0) |
| Extremities AIS, median [IQR] | 2 [0-3] | 0 [0-2] | 2 [0-3] |
| Unknown | 0 (0) | 0 (0) | 0 (0) |
| External AIS, median [IQR] | 0 [0-1] | 0 [0-1] | 0 [0-1] |
| Unknown | 0 (0) | 0 (0) | 0 (0) |

*Abbreviations*: TXA: tranexamic acid; IQR, interquartile range; AIS: abbreviated injury scale; ISS: injury severity score

**Supplemental Figures**

**
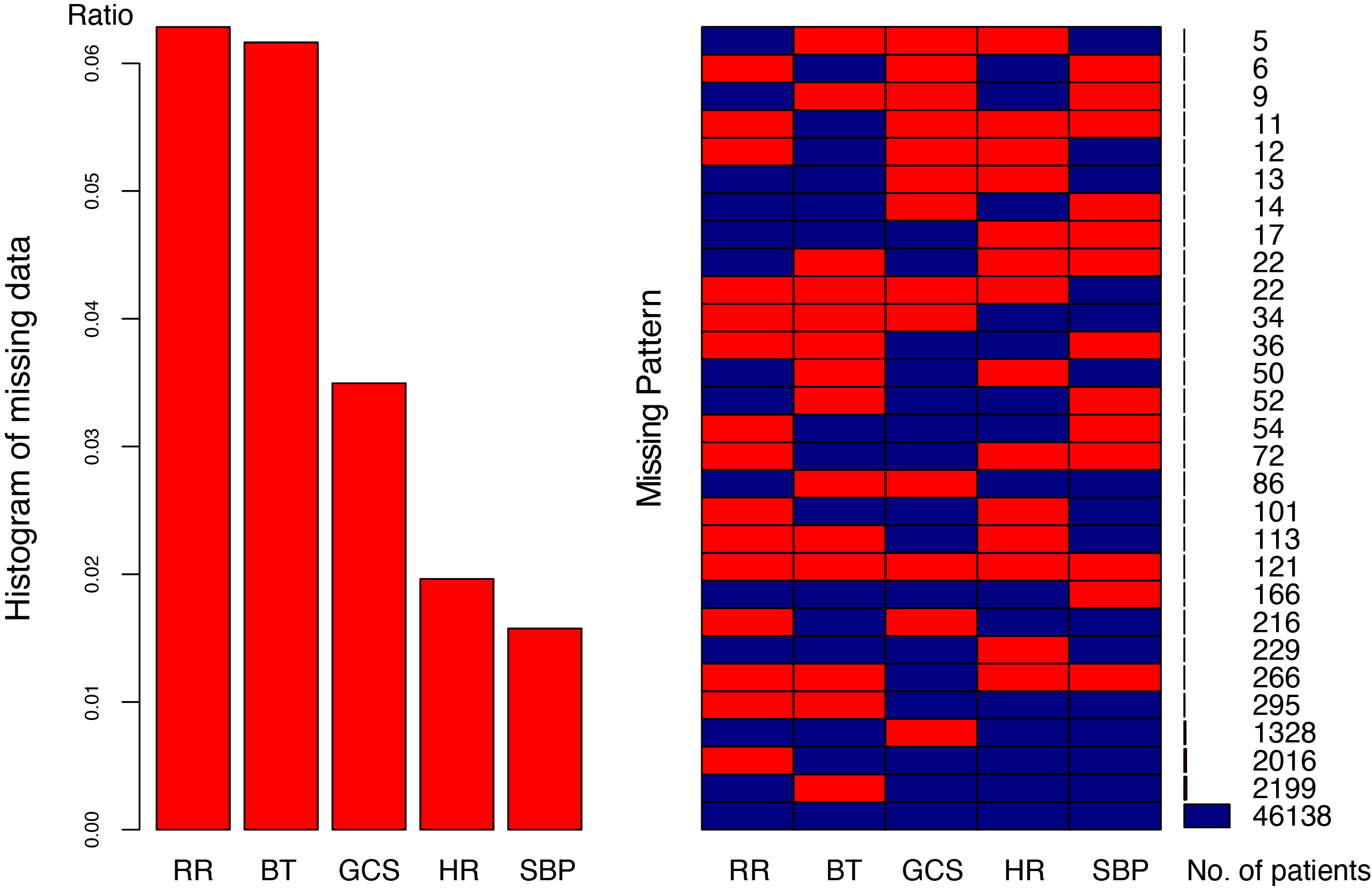
Supplemental Fig. 1 Distribution of missing data**

The histogram on the left shows the proportion of missing values for each variable. The vertical axis represents the percentage of missing values.

The figure on the right illustrates the pattern of missing values. Red cells indicate missing values, while blue cells signify the presence of data. The numerical labels on the right side of the figure represent the number of patients.

*Abbreviations*: RR, respiratory rate; BT, body temperature; GCS, Glasgow Coma Scale; HR, heart rate; SBP, systolic blood pressure.

**Supplemental Fig. 2 Convergence plots for each variable with multiple imputation**

*Abbreviations*: RR, respiratory rate; HR, heart rate; SBP, systolic blood pressure; GCS, Glasgow Coma Scale; BT, body temperature.

**Supplemental Fig. 3 Distribution of variables after multiple imputation
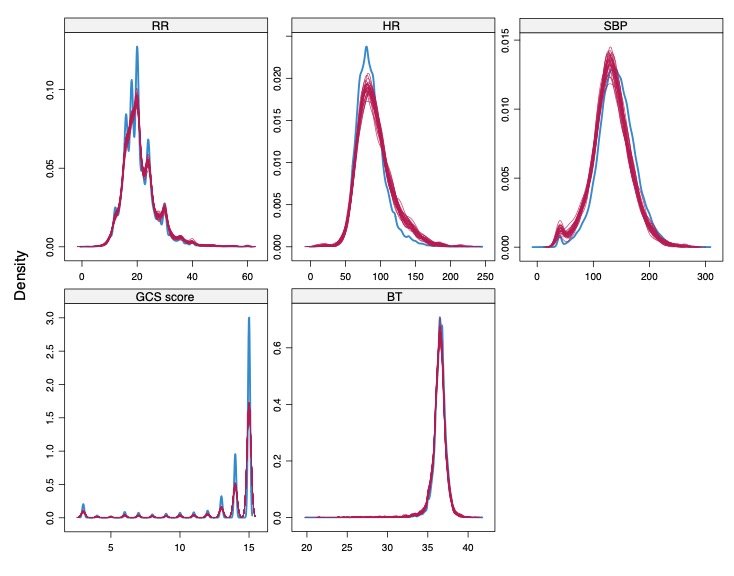
**

The blue line represents the naïve data, while the red line indicates the data after imputation of missing values.

*Abbreviations*: RR, respiratory rate; HR, heart rate; SBP, systolic blood pressure; GCS, Glasgow Coma Scale; BT, body temperature

**Supplemental Fig. 4 Heatmap of VIF between clinical variables for phenotyping**

*Abbreviations*: VIF, variance inflation factors; RR, respiratory rate; HR, heart rate; SBP, systolic blood pressure; GCS, Glasgow Coma Scale; BT, body temperature; AIS, Abbreviated Injury Scale.


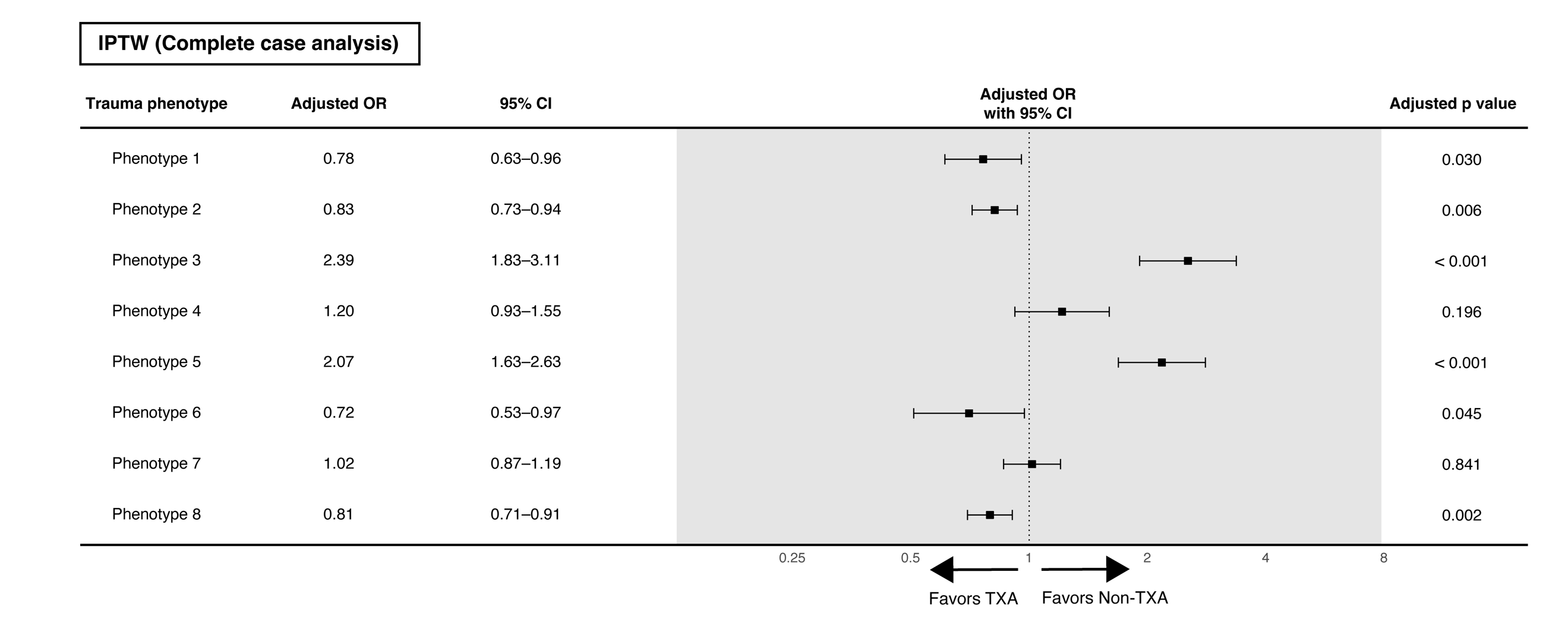
**Supplemental Fig. 5 Analysis using IPTW in complete case**

The above figure shows the results of the analysis in the complete case (n=46,138).

*Abbreviations*: OR, Odds ratio; CI, confidence interval; TXA, tranexamic acid; IPTW, inverse probability treatment weighting.

**Supplemental Fig. 6 Distribution of time from injury to arrival at hospital**

The time from injury to hospital arrival was known for 74.0% (39,739 out of 53,703) of all patients. Of the TXA administration group, 92.8% (5,157 out of 5,559) arrived at the hospital within 120 minutes of injury, and 82.3% (28,118 out of 34,180) of the non-administration group arrived within the same timeframe.

**
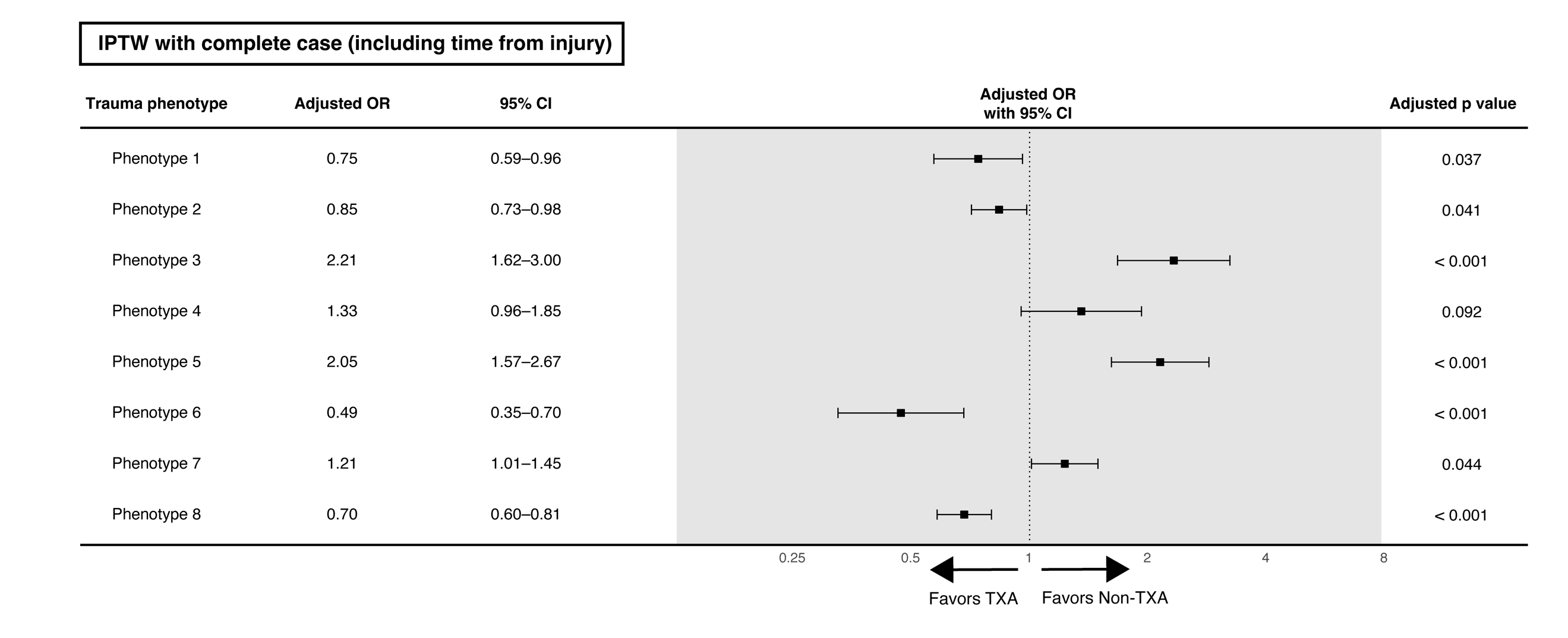
Supplemental Fig. 7 Analysis of the complete data set using IPTW, including the time from injury to transportation**

The figure above presents the analysis results for complete cases (n=34,874) where the duration from injury to transportation is clear.

*Abbreviations*: OR, Odds ratio; CI, confidence interval; TXA, tranexamic acid; IPTW, inverse probability treatment weighting.
